# Lay health coaching intervention for older adults with chronic diseases: study protocol for a pragmatic randomised controlled trial

**DOI:** 10.1186/s13063-024-08649-x

**Published:** 2024-12-18

**Authors:** Edwin K. H. Chung, Eliza Lai-Yi Wong, Hera Hiu-Wah Leung, Dannii Y. Yeung, Eng-Kiong Yeoh, Frank Youhua Chen

**Affiliations:** 1 Department of Management Sciences, City University of Hong Kong, Kowloon Tong, Hong Kong; 2 The Jockey Club School of Public Health and Primary Care, The Chinese University of Hong Kong, Sha Tin, Hong Kong; 3 Department of Social and Behavioural Sciences, City University of Hong Kong, Kowloon Tong, Hong Kong

**Keywords:** Chronic disease, Self-management, Patient activation, Health coaches, Pragmatic, Randomised control trial

## Abstract

**Background:**

A large proportion of older adults suffer from chronic diseases. Health coaching is a promising intervention that enhances individuals’ health knowledge and supports changes in health behaviours. Even though health professionals usually conduct health coaching interventions, lay health workers from different backgrounds account for a growing segment of health coaches over the years. The planned study’s main objective is to investigate whether health coaching by lay health workers is as effective as that by health professionals.

**Methods:**

The effects of health coaching intervention by lay health workers will be examined in comparison with that by health professionals within a single-blind, multi-centre, randomised controlled trial with a follow-up assessment after 3 months. A total of 380 community-dwelling older adults with chronic diseases will be recruited and randomly assigned using a 1:1 ratio into the intervention and control groups. The intervention group will receive a 3-month health coaching intervention delivered by lay health workers, whereas the control group will receive the intervention delivered by health professionals. Primary outcomes include patient activation, physical activity and nutrition behaviours.

**Discussion:**

The expected findings of this study will advance the health coaching literature, research and practice by determining whether health coaching by lay health workers is as effective as that by health professionals in enhancing older adults’ knowledge, skills and confidence in chronic disease self-management and promoting changes in health behaviours. If proven effective, the inclusion of lay health workers in delivering effective self-management interventions should be advocated to reduce the over-reliance on health professionals in the primary healthcare system.

**Trial registration:**

ISRCTN, ISRCTN73836238. Registered 8 November 2023.

**Supplementary Information:**

The online version contains supplementary material available at 10.1186/s13063-024-08649-x.

## Administrative information

Note: The numbers in curly brackets in this protocol refer to SPIRT checklist item numbers. The order of the items has been modified to group similar items (see https://www.equator-network.org/reporting-guidelines/spirit-2013-statement-defining-standard-protocol-items-for-clinical-trials/).

**Table:** 

Title {1}	Lay health coaching intervention for older adults with chronic diseases: study protocol for a pragmatic randomised controlled trial
Trial registration {2a and 2b}	ISRCTN registry ISRCTN73836238. The first submission date is 19 October 2023.
Protocol version {3}	The current protocol is version dated on 22 July 2024.
Funding {4}	This study is supported by the Bank of China (Hong Kong) Limited (Internal code: 9229042).
Author details {5a}	**Edwin K. H. CHUNG** e.chung@cityu.edu.hk Department of Management Sciences, City University of Hong Kong, Kowloon Tong, Hong Kong SAR **Eliza Lai-Yi WONG (Corresponding Author)** lywong@cuhk.edu.hk The Jockey Club School of Public Health and Primary Care, The Chinese University of Hong Kong, Sha Tin, Hong Kong SAR **Hera Hiu-Wah LEUNG** herleung@cityu.edu.hk Department of Management Sciences, City University of Hong Kong, Kowloon Tong, Hong Kong SAR **Dannii Y. YEUNG** dannii.yeung@cityu.edu.hk Department of Social and Behavioural Sciences, City University of Hong Kong, Kowloon Tong, Hong Kong SAR **Eng-Kiong YEOH** yeoh_ek@cuhk.edu.hk The Jockey Club School of Public Health and Primary Care, The Chinese University of Hong Kong, Sha Tin, Hong Kong SAR **Frank Youhua CHEN (Corresponding Author)** youhchen@cityu.edu.hk Department of Management Sciences, City University of Hong Kong, Kowloon Tong, Hong Kong SAR
Name and contact information for the trial sponsor {5b}	Bank of China (Hong Kong) Limited Address: Bank of China Tower, 1 Garden Road, Hong Kong SAR Email: corp_comm@bochk.com
Role of sponsor {5c}	The funder is not involved in the study design, data collection, analysis, interpretation of data, the writing of this article, or the decision to submit it for publication.

## Introduction

### Background and rationale {6a}

Due to decreased birth and mortality rates, the global population aged 60 years and above is projected to increase from 10% in 2022 to 16% in 2030 [1, 2]. With the upward shift in age distribution, the prevalence of chronic diseases is increasing. More than half of the adult population worldwide aged 60 years and above suffers from two or more chronic diseases, such as hypertension, diabetes and heart disease [3]. Previous studies show that individuals with chronic diseases are often associated with increased depression and decreased quality of life, particularly amongst those with poor control of health risk factors and comorbidities [4, 5]. Furthermore, chronic diseases place a substantial financial strain on the healthcare system. For instance, the total cost in the United States for the direct treatment of chronic diseases was more than $1 trillion in 2016 [6]. A systematic review of the financial burden related to chronic diseases indicated that chronic diseases accounted for at least 25% of healthcare budget in the European Union between 2008 and 2018 [7]. Therefore, with the increasing number of older adults with chronic diseases and the associated costs to the healthcare system, effective self-management of chronic diseases amongst older adults becomes increasingly important to alleviate financial strain on the healthcare system and ensure the quality of their prolonged lifespan [8].

In the past two decades, health coaching has emerged as an often-espoused and promising intervention to support individuals living with chronic diseases in setting self-care goals and initiating behavioural changes to improve health outcomes and quality of life [9–11]. Broadly defined, health coaching is patient-centred, focusing on patient-determined goals, incorporating self-discovery and active learning, encouraging accountability for behavioural goals and providing some health education alongside the coaching. It is facilitated by someone who is trained in behavioural change, communication and motivational interviewing skills [12]. Through a collaborative partnership with health coaches, health coaching aims to enhance patients’ medical and self-care knowledge, self-efficacy in chronic disease management and engagement in a healthy lifestyle [13, 14]. Although evidence shows the effectiveness of health coaching in increasing self-efficacy [15, 16] and improving health behaviours, such as physical activity, weight control and medication adherence [17–20], how different intervention components, such as the professional experience of health coaches and modalities of health coaching (e.g. face-to-face, telephone or electronic), are necessary for its effectiveness remains to be understood. Such a study could provide evidence of the efficacy of health coaching across different types of coaching [21] and help standardise health coaching interventions and optimise their effectiveness [11, 22]. For example, a systematic review evaluating the modalities of pharmacist health coaching found that favourable clinical outcomes were reported in each of the 12 reviewed studies regardless of the health coaching modality [23]. This finding suggested that no superior modality of pharmacist health coaching. To further explore the effects of intervention components, the present study therefore aims to examine how different types of health coaches may influence the effectiveness of health coaching.

In health coaching, health coaches take on the role of improving patients’ health literacy and guiding them to set and achieve realistic health goals through educational materials, patient-centred communication and empowerment [24, 25]. On the basis of their clinical experience, health coaches can be broadly categorised into two groups: health professionals and lay health workers [22]. Health professionals encompass various clinical practitioners, such as physicians, nurses, dieticians, pharmacists and students in these fields. By contrast, lay health workers usually do not possess specified credentials or regulated professional status. Examples include certified health coaches, health promotion workers and graduates of health-related fields (e.g. public health, nutrition and physiology). With profound clinical experience in treating chronic diseases and knowledge of medication management, health professionals are primarily considered to fit the role of health coaches [26, 27]. However, lay health workers from various backgrounds account for a growing segment of health coaches over the years [10, 28, 29]. In many low- and middle-income countries, lay health workers are increasingly recognised for their role in improving health equity for marginalised groups [30]. They tend to share similar values, socioeconomic status and/or cultural backgrounds with patients [31], and their interactions with patients are seen as non-hierarchical and less stigmatising than those with health professionals [32, 33]. Thus, patients are more inclined to engage with a health coach who is a lay health worker, particularly when the health coaching intervention is community-based.

Although lay health workers are increasingly taking up the role of health coaches [12], early systematic reviews of health coaching often purposefully exclude interventions delivered by lay health workers and focus mainly on studies published in English [20, 34]. Therefore, some researchers have advocated for paying more attention to the effectiveness of lay health coaching [21]. Few studies of lay health coaching have shown positive effects on self-management of chronic conditions (e.g. glycaemic and blood pressure control) and behavioural changes (e.g. physical activity and nutrition behaviours) [10, 35, 36]. For example, Willard-Grace et al. [10] found that a 9-month lay health coaching has improved inhaler adherence and technique amongst middle-aged and older American patients with moderate to severe chronic obstructive pulmonary disease (COPD). However, similar to health coaching delivered by health professionals [34], previous systematic reviews of lay health coaching for diabetes found that lay health workers varied in their ways of supporting, educating, advocating for and facilitating patients’ self-management [37, 38], resulting in disparate findings. For example, Dye et al. [39] did not find significant differences in emergency care use and hospital admission rates between lay health coaching and control groups in a small sample of older patients with cardiovascular diseases, congestive heart failure and diabetes. Therefore, whether health coaching delivered by lay health workers is as effective as that delivered by health professionals requires further examination, especially when the design of health coaching intervention (e.g. target population, intervention duration, and method of delivery) varies across studies.

This study will pay particular attention to the effects of lay health coaching on community-dwelling older adults with diverse chronic diseases. Previous health coaching studies were often conducted on relatively younger patients, with at least half of the participants being under 60 years [16, 40–42]. Whether health coaching is still effective in older adults, particularly those with low educational attainment, remains uncertain. Furthermore, health coaching studies have often focused on specific diseases such as diabetes and COPD [16, 41]. Consequently, the findings of these studies may not be generalisable to older adults with multimorbidity, which constitutes over 50% of the older population worldwide [3]. For example, by using trials within cohort design, Panagioti et al. [43] found that although older adults with multimorbidity participating in a 6-month telephone health coaching demonstrated lower levels of emergency care use, their patient activation, quality of life and risk of depression did not significantly improve compared with those with only usual care. To address these knowledge gaps, the present study adopts a randomised controlled trial (RCT) design to compare the effects of health coaching interventions delivered by lay health workers and health professionals in community-dwelling older adults with diverse chronic diseases.

### Objectives {7}

This study aims to (1) examine the effects of health coaching in community-dwelling older adults with various chronic diseases, and (2) compare the effects of two types of health coaches (i.e. health professionals and lay health workers) on health coaching outcomes. The effectiveness of health coaching is often measured through individual changes in health knowledge and behaviours, psychological status and clinical outcomes [36, 44]. With reference to previous health coaching studies [40, 43, 45, 46], the present study will measure patient activation, physical activity and nutrition behaviours as primary outcomes and depressive symptoms, body mass index (BMI) and systolic and diastolic blood pressure as secondary outcomes. This study hypothesised that the primary and secondary outcomes will be improved after participation in health coaching, and the improvements in health coaching delivered by lay health workers are comparable to those delivered by health professionals.

### Trial design {8}

This study is a single-blind, multi-centre RCT with a two-group pre-test and post-test between-groups design, in accordance with SPIRIT guidelines [47]. It delivers a 3-month health coaching intervention either by lay health workers (intervention group) or health professionals (comparison group) to community-dwelling older adults with chronic diseases. Survey measurements of primary and secondary outcomes will be taken before and immediately after the intervention. The study will compare the effects of the intervention and control groups at 3 months.

## Methods: participants, interventions and outcomes

### Study setting {9}

The participants are older adults with chronic diseases from a large community setting in Hong Kong.

### Eligibility criteria {10}

Research assistants will assess the eligibility of interested participants. The inclusion criteria are Chinese adults (1) aged 60 years and above, (2) who live in the community and (3) who are diagnosed with at least one of the following chronic diseases or conditions: hypertension, hyperlipidaemia, diabetes, heart disease, COPD, chronic pain, elevated blood pressure or central obesity. To ensure capabilities in communication and decision-making for daily living, the exclusion criteria are set as follows: (1) have impaired cognitive function (a score ≤ 16th percentile in Montreal Cognitive Assessment 5-min) [48]; (2) have poor functional mobility (Timed Up and Go test ≥ 30 s) [49]; (3) are diagnosed with mental disorders (e.g. schizophrenia or depression) and/or dementia; (4) are not able to communicate in Cantonese, Mandarin, and English; and (5) have severe hearing problem that cannot be corrected by hearing aids.

### Who will take informed consents? {26a}

Consent procedures were approved by the Human Subjects Ethics Sub-committee of City University of Hong Kong. Eligible participants will receive a consent form with a summary of the study, including study procedures, risks and benefits, intervention content, voluntary participation in the study, how confidentiality will be maintained and an explanation of data collection and sharing procedures. They will be asked to provide a written informed consent by signing their names.

### Additional consent provisions for collection and use of participant data and biological specimens in ancillary studies {26b}

No additional consent provisions are needed for collecting or using biological samples. If an ancillary study will be conducted in the future, prior consent of participants to be re-contacted and informed consent will be taken for the ancillary study following approval from the Human Subjects Ethics Sub-committee of City University of Hong Kong.

### Interventions

#### Explanation for choice of comparators {6b}

Health coaching recognises the importance of individualised guidance and support in managing the self-care tasks associated with chronic disease conditions, including medication adherence, physical activity, dietary modifications and health monitoring. Whilst evidence shows the effectiveness of health coaching, most interventions are delivered by health professionals [16, 45, 50]. Whether health coaching delivered by lay health workers is still effective in enhancing individuals’ self-management competence and promoting healthy behavioural changes, particularly for community-dwelling older adults, remains uncertain. Therefore, two health coaching interventions will be compared: one delivered by lay health workers (intervention group) and another delivered by health professionals (comparison group).

#### Intervention description {11a}

The comparison and intervention groups will be assigned to a health coaching intervention. The comparison group will receive a health coaching intervention delivered by health professionals, such as nurses and dieticians, to promote a healthy lifestyle and self-management of chronic diseases. The intervention group will receive the same health intervention, except that it is delivered by lay health workers, including health promotion workers and graduates in health studies. Thus, the procedure and format of health coaching interventions remain the same across comparison and intervention groups whilst the clinical knowledge and experience of health coaches vary between them.

The health coaching intervention implemented in this study is based on effective health coaching components (e.g. motivational interviewing techniques and solution-focused goal setting) identified in systematic reviews and studies of health coaching in chronic disease management [34, 43, 51, 52]. All health coaches must complete over 100 h of training in a health coaching curriculum [53], supplemented with chronic disease-specific content (e.g. symptoms and regimens). The curriculum covers active listening and non-judgmental communication, navigating healthcare systems, setting self-management goals and motivational interviewing techniques. Although older adults may have different chronic diseases, they face similar challenges in managing their health conditions [54]. The chronic disease-specific content thus covers guidance for medication adherence, physical activity, dietary modifications and health monitoring. Due to the pragmatic nature of the study, further supervision processes will not be possible. Similar to previous studies, the health coaching intervention proceeds for 3 months [55, 56], and health coaches provide one-on-one support to older adults with chronic diseases, with a maximum caseload of 20 older adults at any given time. There will be a minimum frequency defined as one in-person or telephone contact every 6 weeks.

The health coaching intervention begins with an in-person goal-setting session at participants’ homes. During this session, participants will be presented with an individual report summarising their health status as assessed before the intervention. This report serves as a starting point for discussion. Participants are encouraged to establish one or more personally meaningful goals to work toward during the intervention. Action plans, health-related goals (e.g. lowering blood pressure) and strategies are generated by participants themselves rather than imposed by the health coaches. Subsequent sessions, either in person or over the phone, are designed to support participants’ self-determined goals by building skills in problem solving, reacting to problems, stress management, communication strategies and maintaining healthy habits. The interaction between participants and health coaches will be documented, including the date, time and relevant notes.

#### Criteria for discontinuing or modifying allocated intervention {11b}

Given that the procedures in this study are generally low risk, the likelihood of participants’ withdrawal or discontinuation is minimal. However, since the participants are older adults with chronic diseases, the chance of dropping out due to sudden health deterioration is still possible. The participants will be able to discontinue the intervention sessions at any point at their request.

#### Strategies to assess and improve adherence to interventions {11c}

The participants will receive phone reminders about the date and time of the health coaching session. They are encouraged to stay for the entire intervention.

#### Relevant concomitant care permitted or prohibited during the trial {11d}

Participants will be excluded if they have enrolled in a concurrent lifestyle intervention research study during this study. However, participants can receive concomitant treatment (e.g. usual care, psychotherapy and pharmacotherapy), which will be assessed at follow-up.

#### Provisions for post-trial care {30}

No provision for post-trial care will be made because the interventions have been found to be safe. After completing the study, participants will return to their usual care.

### Outcomes {12}

Table 1 displays the SPIRIT flow diagram for the schedule of enrolment, intervention and assessment. Specifications of measures and data collection are provided below.

**Table 1:**
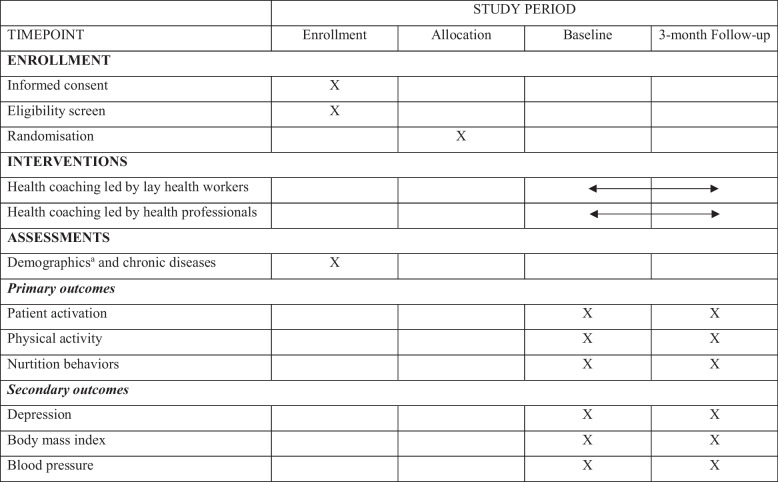
SPIRIT flow diagram of the schedule of enrollment, intervention and assessment of the study

^a^Demographic variables include age, sex, education and number of chronic diseases

#### Patient activation

The 13-item Chinese version of the short-form Patient Activation Measure (PAM-13) [57, 58] will be used to assess patient knowledge, skills and confidence in self-management for chronic conditions. Sample items include ‘I am confident that I can follow through on medical treatments I may need to do at home’ and ‘I am confident that I can figure out solutions when new problems arise with my health.’ Each item is rated on a four-point Likert scale (1 = *strongly disagree* to 4 = *strongly agree*). The item response total will be transformed into an activation score ranging from 0 to 100 using the scale developers’ established algorithm [58], with a higher score denoting a high level of patient activation. A research license to use this scale will be obtained from Insignia Health, University of Oregon.

#### Physical activity and nutrition behaviours

The physical activity and nutrition subscales of the validated Chinese version of the short-form Health-Promoting Lifestyle Profile-II (HPLP-II) [59], (Walker S, Hill-Polerecky D: Psychometric evaluation of the health-promoting lifestyle profile II, Unpublished) will be adopted to assess participants’ engagement in physical activity (e.g. ‘do stretching exercises’) and nutrition behaviours (e.g. ‘eat 3–5 servings of vegetables each day’). Each item is measured using a four-point Likert scale (1 = *never* to 4 = *always*), with higher scores indicating more frequent engagement in physical activity and nutrition behaviours, respectively.

### Secondary outcomes

#### Depressive symptoms

The depressive symptoms will be measured by the 4-item short form of the Geriatric Depression Scale (GDS-4) [60]. Each item is rated using a binary scale (0 = *no* and 1 = *yes*). A higher summative score indicates more depressive symptoms in the past week.

#### Body mass index

The participants’ weight will be measured on the scale in light clothing, without shoes, and with a precision of 0.1 kg. Height will be measured to the nearest 0.1 cm by using a stadiometer with the stretch stature method and then converted to metres. BMI will be calculated using the standard formula (weight in kg)/(height in metres)^2^.

#### Blood pressure

Participants will be asked to rest for at least 15 min before blood pressure measurements are taken. Systolic and diastolic blood pressure will be measured twice, at a 2-min interval, on the left arm in a sitting position using an Omron HEM-7130 model automatic digital blood pressure monitor (Omron Healthcare, Kyoto, Japan). Each participant will be seated upright with his or her left arm supported at heart level. The mean of the first and second systolic and diastolic measurements will be reported as the blood pressure values for individual patients if the difference is within 5 mmHg. If the readings differ by more than 5 mmHg, the measurements will be repeated.

#### Participant timeline {13}

**Fig. 1:**
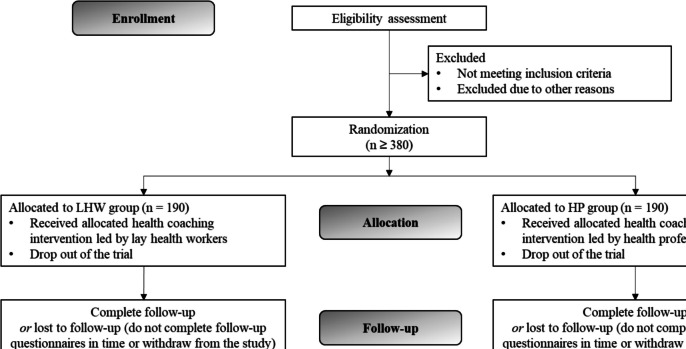
Flow chart of the study design

The participants’ flow for the trial is outlined in Fig. 1. Baseline data, including demographics and chronic diseases, will be collected in the pre-screening assessment. Eligibility of interested participants will be assessed by research assistants. Eligible participants will be randomly assigned to either one of the two arms (intervention or comparison group) and will subsequently be asked to complete the baseline and 3-month follow-up assessments.

#### Sample size {14}

The required sample size is estimated for one of the primary outcomes, i.e. patient activation. With reference to previous studies of patient activation interventions on chronic disease self-management [43, 61], the sample size is estimated on the basis of an effect size *f* = 0.2. Using G*Power, power analyses revealed that a total sample size of 266 is required to detect differences amongst the two intervention conditions (i.e. health coaching delivered by lay health workers vs. health professionals), using a multivariate analysis of variance with an effect size of 0.2, a power of 90% and an alpha level of 0.05, with 133 individuals in each condition. Considering the dropout rate of 30% over a period of three months, the target sample size is adjusted to 380 (with 190 individuals in each condition).

#### Recruitment {15}

The study protocol was approved by the Human Subjects Ethics Sub-committee of City University of Hong Kong. Older Chinese adults with chronic diseases will be invited through the centres of collaborating non-governmental organisations in different Hong Kong clusters. The study team will answer any questions raised and will ask for written informed consent if the older adult is eligible.

### Assignment of interventions: allocation

#### Sequence generation {16a}

Eligible participants will be randomised to either intervention or comparison groups on a 1:1 allocation ratio by using the R package *randomizr* [62] for R software (version 4.3.3). Four stratification factors, each with two levels, will be used: age (< 75 years vs. ≥ 75 years), sex, education (primary and below vs. secondary and above) and number of chronic diseases (≤ 2 vs. > 2). This stratification helps to reduce between-group differences at baseline and increase statistical power with a fixed sample size [63].

#### Concealment mechanism {16b}

Participants are randomised by an independent researcher by using R software, following a password-protected list with the allocation results. The allocation results will only be accessed by authorized study staff and the study coordinator upon randomisation by the independent researcher. The research assistants conducting baseline and follow-up assessments will not be informed about the allocation results.

#### Implementation {16c}

After obtaining written informed consent and collecting baseline data, an independent researcher will generate the allocation and inform the study coordinator about the results. To keep the research assistants blind to the allocation, the study coordinator will directly inform the health coaches about the list of assigned participants.

### Assignment of interventions: blinding

#### Who will be blinded {17a}

Research assistants, participants and health coaches are kept blind to the treatment allocation. The study coordinator will not be blinded but is strongly instructed not to disclose the assigned treatment during the study period.

#### Procedure for unblinding if needed {17b}

Not applicable. There is no need to unblind the research assistants, participants or health coaches.

### Data collection and management

#### Plan for assessment and collection of outcomes {18a}

Baseline and 3-month follow-up assessments will be conducted by trained research assistants to assess the primary and secondary outcomes. A detailed description of the instruments used in the study can be found in the ‘Outcomes {12}’ section. Data will be collected through the online survey platform QuestionPro to minimise data entry errors. All data will be anonymised and saved in a password-protected database on a firewall-protected local network server at City University of Hong Kong. Access to all data will be restricted to authorised study staff. The data will be stored, coded and cleaned in R software before analyses (e.g. checking for duplicates, coding of missing data and date of entry).

#### Plan for promote participant retention and complete follow-up {18b}

The health coaching sessions will be arranged between health coaches and participants upon mutual agreement. The participants will receive reminders from the health coaches regarding the day and time of the health coaching session. To maximise retention, the health coaching sessions will be conducted in participants’ homes or via telephone. Research assistants will follow-up with and motivate the participants for the 3-month follow-up assessment.

#### Data management {19}

Each participant will be assigned a unique participant identification code. All participants’ personal data will be encrypted with the identification codes. Demographic information linking the participants to the data will be stored in a separate file. All data will be entered into a password-protected database and managed on a firewall-protected local network server at City University of Hong Kong. Access to all data will be restricted to authorised study staff.

#### Confidentiality {27}

Identifiable individual participant information obtained in this study is confidential, and disclosure to non-relevant third parties is prohibited. The final dataset will contain only re-identifiable information (i.e. a unique participant identification code) and anonymised data entries. All publications associated with the results of the study will involve de-identified data, ensuring the confidentiality of participants.

#### Plans for collection, laboratory evaluation, and storage of biological specimens for genetic or molecular analysis in this trial/future use {33}

Not applicable. No biological specimens will be collected nor stored in this study.

## Statistical methods

### Statistical methods for primary and secondary outcomes {20a}

Descriptive statistics will be used to summarise participants’ baseline demographics and outcome variables throughout the study time points. Attrition analysis and randomisation tests will be carried out to test for differences in demographic and outcome variables between the intervention and comparison groups. Repeated measures ANCOVA will be conducted to examine any significant improvements in the primary and secondary outcomes across baseline and 3-month follow-up assessments whilst controlling for the effects of demographics (i.e. age, sex, education, chronic diseases) and the frequency of intervention sessions. Generalised estimating equation (GEE) analyses will be conducted to compare the primary and secondary outcomes at the 3-month follow-up between the intervention and comparison groups whilst controlling for the effects of demographics and the frequency of intervention sessions. The intention-to-treat principle will be adopted in the analyses for all the primary and secondary outcomes by imputing missing follow-up outcome data with their baseline values (i.e. assuming no change after the intervention for the primary and secondary outcomes) under the assumption of data being missing not at random. This assumption is plausible in the sense that missing data because of dropouts or non-responses are more likely amongst old-old participants with severe health problems. All statistical analyses will be conducted using R software. The treatment effect within and between the two groups will be assessed using Cohen’s *d* statistic [64].

### Interim analyses {21b}

Not applicable. No interim analysis will be conducted because the adverse effects of participation for participants are minimal in this study.

### Methods for additional analyses (e.g. subgroup analyses) {20b}

To evaluate the robustness of the analysis results, sensitivity analyses for the primary and secondary outcomes will be conducted under the assumptions that data are missing completely at random (MCAR) and data are missing at random (MAR). Complete case analyses will be conducted for the primary and secondary outcomes. The results are expected to be unbiased under the MCAR condition. Little’s MCAR test will be used to justify the plausibility of the assumption. In addition, if the assumption of MCAR is not supported, the sensitivity analyses will be conducted on the basis of multiple imputations under the assumption of MAR. A subgroup analysis will be conducted to explore any interaction effects.

### Methods in analysis to handle protocol non-adherence and any statistical methods to handle missing data {20c}

Intention-to-treat analysis will be used to minimise the impact of participant dropouts and non-adherence to the protocol by analysing all participants according to their original assignment, regardless of whether they completed the study. Missing data will be imputed using statistical methods to ensure that the analysis is not biased by missing data.

### Plans to give access to the full protocol, participant-level data, and statistical code {31c}

The full protocol will be accessible after publication. Upon reasonable request, the corresponding authors will be able to provide an anonymised dataset of participant-level data and statistical code after the main results are published.

### Oversight and monitoring

#### Composition of the coordinating centre and trial steering committee {5d}

The study team is responsible for monitoring the integrity of the study and participant safety. The study team will oversee the consent procedures, safety plans prior to the study commencement and study progress in terms of participant recruitment and retention, adverse events and protocol adherence.

#### Composition of the data monitoring committee, its role and reporting structure {21a}

The committee comprises the principal investigator, co-investigators, a project manager and a data manager. The project manager is responsible for adherence to data collection operating procedures. The data manager monitors data collection and raises queries with the project manager during data monitoring. The data manager prepares data exports for weekly review and reports to the principal investigator and co-investigators upon request.

#### Adverse event reporting and harms {22}

Adverse events are any undesirable experiences that occur on a participant during the study, regardless of whether they are related to the study procedures. The project manager of the study will be notified within 12 h if a research staff member or health coach witnesses or becomes aware of a participant reporting an adverse event. Participants will be asked about the occurrence of adverse events in the follow-up assessment immediately after the intervention. If a serious adverse event occurs, the project manager will notify the corresponding centres of collaborating non-government organisations within 48 h to ensure the safety of participants.

#### Frequency and plans for auditing trial conduct {23}

Study activities related to consent, randomisation, data collection and intervention will be monitored on an ongoing basis. The data management team will monitor the data on a firewall-protected local network server at City University of Hong Kong. The data manager will upload outcome data to the server monthly.

#### Plans for communicating important protocol amendments to relevant parties (e.g. trial participants, ethical committees) {25}

If there are any major protocol modifications to the protocol, the team will communicate the changes to the investigators, the Human Subjects Ethics Sub-committee, study participants, trial registries and journals.

#### Dissemination plans {31a}

The results of this study will be disseminated via peer-reviewed journal articles and presentations at international conferences.

## Discussion

The increasing prevalence of chronic diseases in older adults poses significant challenges not only to individual well-being but also to the primary healthcare system. Effective self-management interventions to enhance older adults’ health knowledge and support changes in health behaviours are important for aging-in-place. This study protocol, therefore, describes the design of an RCT for the lay health coaching intervention. The focuses of the study are to examine whether the beneficial effects of health coaching can be extended to older adults with diverse chronic diseases in a community setting and whether lay health workers are as effective as health professionals in delivering health coaching intervention. To the best of the authors’ knowledge, this study is the first study to directly compare the treatment effects of health coaching delivered by lay health workers and health professionals.

The health coaching intervention in this study takes into account the individual courses of chronic diseases and the emergence and severity of comorbidities and provides a tailored approach to support lifestyle changes. By demonstrating the effectiveness of lay health coaching, this study can empower older adults to take an active role in managing their chronic conditions, fostering their self-efficacy and resilience in the face of age-related deterioration [39]. Furthermore, echoing the recommendations of embracing a diverse set of healthcare providers to improve access to healthcare services [65], this study is dedicated to examining whether lay health workers from diverse backgrounds can provide self-management support as effectively as health professionals. The positive findings of this study will provide evidence for integrating lay health workers into the healthcare system to improve the inclusivity and accessibility of healthcare services, especially for marginalised populations [30]. It will also encourage a collaborative model of chronic disease management where health professionals focus on more complex medical issues of the patients whilst lay health workers address their lifestyle and behavioural changes, thus reducing the power imbalances in favour of health professionals [65] and alleviating the pressure of professional personal shortage in chronic disease management. Furthermore, the potential to standardise health coaching interventions can lead to more uniform practices across different healthcare settings, improving consistency and quality of care.

## Trial status

The trial is currently operating under protocol version 1.0, and the first participant was recruited on 2 January 2024. It is expected to recruit 5 to 10 eligible participants each week; thus, recruitment is expected to be completed in December 2024. The expected completion of the project, including all follow-up appointments, is March 2025.

## Supplementary Information

Supplementary Material 1.

## Data Availability

The data and materials will be available after the main results have been published.

## Abbreviations

BMI: Body mass index
COPD: Chronic obstructive pulmonary disease
GEE: Generalised estimating equation
GSD-4: Geriatric Depression Scale
HPLP-II: Health-Promoting Lifestyle Profile-II
MAR: Missing at random
MCAR: Missing completely at random
PAM-13: Patient Activation Measure
RCT: Randomised controlled trial
